# Multidrug-Resistant Shiga Toxin–Producing *Escherichia coli* O118:H16 in Latin America

**DOI:** 10.3201/eid0908.030062

**Published:** 2003-08

**Authors:** Antonio Fernando Pestana de Castro, Beatriz Guerra, Luciana Leomil, Leila Aidar-Ugrinovitch, Lothar Beutin

**Affiliations:** * Universidade de São Paulo, São Paulo, Brazil; † Federal Institute for Risk Assessment, Berlin, Germany; ‡ Universidade de Campinas, Campinas, Brazil; § Robert Koch-Institute, Berlin, Germany

**To the Editor:** We report the first isolation of a multiple antimicrobial drug–resistant strain of Shiga toxin–producing *Escherichia coli* (STEC) O118:H16 from cattle in Latin America. The strain was isolated during a study of fecal STEC in 205 healthy and 139 diarrheic cattle on 12 beef farms in the state of São Paulo, Brazil, in February 2000; one case of STEC was found in a 1-month-old calf with diarrhea. This bovine STEC O118:H16 strain showed resistance to eight antimicrobial substances; the following resistance (R)-genes were detected: ampicillin (*bla*_TEM1-like_), kanamycin and neomycin (*aphA1*), streptomycin (*strA/B*), sulphametoxazol (*sul2*), tetracyclin (*tet*[A]), trimethoprim (no *dfrA1,* *A5*, *A7*, *A12*, *A14*, or *A17*), and trimethoprim/sulphamethoxazol. The STEC O118:H16 strain from Brazil was found to be similar for virulence genes (Shiga toxin 1 [*stx*1], intimin beta 1 [*eae* β1], and EHEC-hemolysin [E-*hly*A]) and for antimicrobial drug resistance to STEC O118:H16 strains, which were isolated in different countries of Europe (*1*). Beginning in 1986, STEC O118:H16 was identified as an emerging pathogen for calves and humans in Belgium and Germany (*2*–*4*). Cattle and human STEC O118:H16 isolates were similar in virulence attributes and antimicrobial drug resistance and belonged to a distinct genetic clone (*1*). Transmission of these pathogens from cattle to humans on farms was observed (*5*).

Beginning in 1996, STEC O118:H16 has become important as an emerging pathogen in humans and has been associated with bloody diarrhea and hemolytic uremic syndrome (*2*). Analysis of the antimicrobial resistance profiles showed that >96% of the European STEC O118:H16 strains showed resistance to one or more antimicrobial drugs in contrast to the 10% to 15% drug-resistant strains that were detected among STEC belonging to other serotypes (*1*,*6*,*7*). STEC O118:H16 showing multiresistance in up to eight different antimicrobial drugs predominated among younger isolates, indicating that drug resistance genes have accumulated over time in STEC O118:H16 strains. The frequency of antimicrobial drug resistance in STEC and Stx-negative *E. coli* in humans and animals was compared in a study by Schroeder et al. (*8*). Among human clinical *E. coli* isolates, antimicrobial resistance was less frequently observed in STEC than in Stx-negative strains, whereas in cattle, antibiotic-resistant strains were found at similar frequencies in both groups of *E. coli*. The relatively higher frequency of antimicrobial-resistant STEC in cattle was explained by the use of antimicrobial drugs in cattle production, whereas human infections with STEC are generally not treated with antibiotics (*8*). Cattle could thus be an important source of new emerging antibiotic-resistant STEC strains such as O118:H16.

The genetic basis of antimicrobial resistance in STEC O118:H16 is broad, including R-plasmids, integrons, transposons, and chromosomally inherited drug-resistance genes. Fluoroquinolone resistance has also been acquired by some STEC O118:H16 strains (*1*). The heterogenicity of antimicrobial drug–resistance patterns, the increase of multidrug-resistant strains over time of isolation, and the evidence for multiple aquisition and genetic location of R-determinants indicate that strains belonging to the STEC O118:H16 clone have a propensity to acquire and accumulate R-genes. The finding that multidrug-resistant STEC O118:H16 is isolated from cattle in South America indicates the global spread of this new emerging EHEC type.
